# Poppers-Induced Methemoglobinemia: A Curious Case of the Blues

**DOI:** 10.7759/cureus.15276

**Published:** 2021-05-27

**Authors:** Daniela M Tello, Andrew V Doodnauth, Krunal H Patel, Diana Gutierrez, Gangacharan R Dubey

**Affiliations:** 1 Internal Medicine, State University of New York (SUNY) Downstate Medical Center, Brooklyn, USA; 2 Pulmonary and Critical Care Medicine, Veterans Affairs New York (VA NY) Harbor Healthcare System, Brooklyn, USA

**Keywords:** methemoglobinemia, hypoxia, jungle juice, poison control, methylene blue

## Abstract

Methemoglobinemia (MetHb) can be a deadly condition at certain levels, presenting in a fulminant form of cyanosis or disguising itself with vague symptoms. Methemoglobinemia is an altered state of the body’s hemoglobin, which can be congenital or acquired. We report a case of a 62-year-old male who presented with altered mental status and hypoxia after consuming “Jungle Juice”, raising concern for methemoglobinemia. A diagnosis of methemoglobinemia was confirmed with arterial blood gas and guidance from New York State poison control. The patient was adequately treated with the antidote methylene blue with a resolution of symptoms. We highlight that methemoglobinemia can present itself in various forms and that early recognition and treatment can prevent fatal outcomes.

## Introduction

Methemoglobinemia (MetHb) can be a deadly condition, presenting in a fulminant form of cyanosis or disguising itself with vague symptoms. MetHb can be congenital or acquired. However, the pathophysiology remains similar. MetHb is oxidized hemoglobin, which changes its heme iron configuration from the ferrous (Fe^2+^) to the ferric (Fe^3+^) state, rendering it incapable of oxygen binding. If severe enough, it leads to tissue hypoxia [1]. Most cases are acquired, resulting from increased methemoglobin formation induced by various exogenous substances. Common causes of methemoglobinemia are ingestion and exposure of the skin or mucous membranes to oxidizing agents such as nitrites [2].

Poppers are inorganic nitrites that have become increasingly popular since the 1970s and are widely available bottled volatile nitrites that are inhaled for recreational purposes for their vasodilatory, muscle-relaxant, and euphoria-inducing properties [3,4].

We report a case of a 62-year-old male who presented to the emergency room with vague symptoms. However, he was saturating at 87% on room air with no cyanosis on physical exam. Upon further review, he reported that he drank a substance minutes beforehand known as “Jungle Juice.” Finally, an arterial blood gas (ABG) confirmed a diagnosis of MetHb.

Upon further investigation and poison control guidance, we discovered that “Jungle Juice” contains acetone and isobutyl nitrite. Our case highlights a known precipitant of methemoglobinemia secondary to the ingestion of a recreational substance, poppers, also known as “Jungle Juice.”

## Case presentation

A 62-year-old male with a past medical history of non-insulin-dependent diabetes mellitus, hyperlipidemia, and atrial flutter, presented to the emergency room with headache, nausea, vomiting, and an unsteady feeling stating he felt like he was going to “pass out.” He reported that his symptoms came on within an hour after drinking approximately 15-30 mL, a substance referred to as “Jungle Juice.”

Vitals upon presentation included a blood pressure of 150/78 mmHg, heart rate of 77 beats/min, respiratory rate of 24 breaths/min, oxygen saturation of 87% on room air, and temperature of 37.1°C. The patient was in no acute distress, alert, and oriented to person, place, and time. No cyanosis was visualized, and a focused neurological exam was unremarkable. The remainder of the physical examination was benign. He was placed on 2L of oxygen via nasal cannula and was admitted to the medical intensive care unit (MICU) for further management.

The complete metabolic panel (CMP) and complete blood count (CBC) were within normal limits. Lactic acid 2.0 (0.5-2.2 mmol/L) and ABG with a pH 7.38 (7.35-7.45), pCO_2_ 37 (35-45 mmHg), HCO3^-^ 22 (22-26 mEq/L), SaO_2_ 93.5 (80-100 mmHg). A serum methemoglobin level > 20%. Electrocardiogram (EKG) showed normal sinus rhythm (NSR), HR 77 bpm, QTc 446 ms, and no ischemic changes identified. The chest radiograph showed no acute process, and the toxicology screen was negative.

We discussed the case with New York State poison control and emergently administered methylene blue infusion at 1 mg/kg over 20 minutes with an adequate response. Methemoglobin levels trended down over the subsequent 24 hours. The patient did not have any signs of organ ischemia and recovered without any acute complications.

## Discussion

Methemoglobinemia is an altered state of the body’s hemoglobin. This new ferric state prevents the oxygen from binding to the hemoglobin, leading to hypoxia of tissues from the decreased oxygen-binding capacity of methemoglobin [1]. It can be congenital or acquired; acquired, either by ingestion, inhalation, or skin exposure [4]. Common medications that can cause methemoglobinemia include local anesthetics like lidocaine, antibiotics including dapsone and sulfa drugs, and nitrites (Table 1) which can absorb through the gastrointestinal tract [4]. Patients can present with cyanosis, dyspnea, tachycardia, lethargy, unresponsiveness, and even death. Two distinct features of methemoglobinemia are cyanosis that does not improve with supplemental oxygen and normal partial pressure of oxygen on arterial blood gas [5].

**Table 1:** Some of the most common causes of acquired methemoglobinemia.

Aniline	Benzocaine	Nitroglycerine	Phenytoin
Rifampin	Arsine	Dapsone	Chloroquine
Naphthalene	Nitrofuran	Clofazimine	Silver Nitrate
Sulfonamides	Phenol	Phenacetin	Bivalent Copper
Alloxan	Exhaust Fumes	Methylene Blue	Bupivacaine Hydrochloride

Symptom severity depends on methemoglobin percentage in the blood. Individuals can be asymptomatic with methemoglobin levels of less than 10%, but cyanosis may be present in MetHb levels above 10% [4]. Above 10%, the blood can look brown or chocolate color [6]. A MetHb level of 20-30% may result in patients experiencing headaches and asthenia, as seen in our patient. Also, at 30-40%, patients experience anxiety, nausea, vomiting, abdominal pain, dyspnea, and chest pain. With MetHb levels around 55%, patients can experience alteration of consciousness and cardiac arrhythmias, and beyond 80% is generally fatal [7].

“Poppers,” also known as “Jungle Juice,” as mentioned by our patient, are volatile nitrites [8]. They have been commonly used for recreational purposes via inhalation since the seventies [9]. “Jungle Juice” is sold in sex shops and is commonly used in men who have sex with men for their vasodilator effects, anal sphincter relaxation, and aphrodisia [3,8,9,10].

The treatment for methemoglobinemia is based on the severity of the symptom. Mild symptoms warrant removing the offending agent, providing high flow oxygen to the patient, and continued monitoring. It can take up to 36 hours for methemoglobinemia to revert to baseline levels [6]. The first-line treatment for methemoglobinemia is methylene blue, which acts as a substrate for nicotinamide adenine dinucleotide phosphate hydrogen (NADPH)-MetHb reductase forming a reduced methylthioninium chloride, an electron donor to reduce Fe^3+^ back to Fe^2+^ [4]. Methylene blue is usually indicated for the treatment of methemoglobinemia when the MetHb level is >30%, or in symptomatic patients (lightheadedness, tachycardia, chest pain, shortness of breath, etc.) regardless of MetHb level [11]. The initial dose of methylene blue is 1 to 2 mg/kg infused over 5 minutes [12,13].

Glucose-6-phosphate-dehydrogenase (G6PD) catalyzes the first step in the pentose-phosphate pathway of glycolysis, leading to reduced glutathione production. In patients with G6PD deficiency, there are low levels of NADPH at baseline. The use of methylene blue in patients with G6PD deficiency is contraindicated because methylene blue facilitates the use of NADPH to reduce methemoglobin back into normal hemoglobin, which may theoretically exacerbate the deficiency in NADPH. Reduced glutathione levels can potentially lead to volatile nitrite-induced hemolysis.

## Conclusions

We presented this case to make evident that methemoglobinemia can present itself in various forms, as seen in our patient disguised among vague symptoms. This case also serves the purpose of making you aware that this can occur after ingestion or inhalation of “Poppers,” commonly sold in sex shops for recreational purposes. Although infrequent, methemoglobinemia can be fatal at blood levels above 80%, which is why physicians must take a thorough history of exposures, medications, and recreational drug use in a patient with unexplained hypoxia. Early recognition and administration of the antidote methylene blue prevent fatal outcomes.
